# Effects of ribavirin on the replication and genetic stability of porcine reproductive and respiratory syndrome virus

**DOI:** 10.1186/s12917-015-0330-z

**Published:** 2015-02-07

**Authors:** Amina Khatun, Nadeem Shabir, Kyoung-Jin Yoon, Won-Il Kim

**Affiliations:** College of Veterinary Medicine, Chonbuk National University Jeonju, Korea, 664-14 Deokjin-Dong 1 Ga, Jeonju, Jeonbuk, 561-756 Republic of Korea; Department of Veterinary Diagnostic and Production Animal Medicine, College of Veterinary Medicine, Iowa State University, Ames, IA USA

**Keywords:** PRRS, Ribavirin, 5-Fluorouracil, 5-Azacytidine and Amiloride

## Abstract

**Background:**

Although modified live virus (MLV) vaccines are commonly used for porcine reproductive and respiratory syndrome virus (PRRSV) control, there have been safety concerns due to the quick reversion of MLV to virulence during replication in pigs. Previous studies have demonstrated that mutant viruses emerged from lethal mutagenesis driven by antiviral mutagens and that those viruses had higher genetic stability compared to their parental strains because they acquired resistance to random mutation. Thus, this strategy was explored to stabilize the PRRSV genome in the current study.

**Results:**

Four antiviral mutagens (ribavirin, 5-fluorouracil, 5-azacytidine, and amiloride) were evaluated for their antiviral effects against VR2332, a prototype of type 2 PRRSV. Among the mutagens, ribavirin and 5-fluorouracil had significant antiviral effects against VR2332. Consequently, VR2332 was serially passaged in MARC-145 cells in the presence of ribavirin at several concentrations to facilitate the emergence of ribavirin-resistant mutants. Two ribavirin-resistant mutants, RVRp13 and RVRp22, emerged from serial passages in the presence of 0.1 and 0.2 mM ribavirin, respectively. The genetic stability of these resistant mutants was evaluated in MARC-145 cells and compared with VR2332. As expected, the ribavirin-resistant mutants exhibited higher genetic stability compared to their parental virus.

**Conclusions:**

In summary, ribavirin and 5-fluorouracil effectively suppressed PRRSV replication in MARC-145 cells. However, ribavirin-resistant mutants emerged when treated with low concentrations (≤0.2 mM) of ribavirin, and those mutants were genetically more stable during serial passages in cell culture.

## Background

Porcine Reproductive and Respiratory Syndrome (PRRS) is one of the most economically important infectious diseases of swine worldwide. The annual loss associated with PRRS to the United States swine industry has been estimated to be approximately 664 million USD [1]. PRRS virus (PRRSV), the etiological agent of PRRS, is classified as a member of the *Arteriviridae* virus family, along with equine arteritis virus (EAV), lactate dehydrogenase-elevating virus (LDV) of mice, and simian hemorrhagic fever virus (SHFV), and belongs to the order *Nidovirales* [2,3]. PRRSV is a small, enveloped virus that contains a single-stranded, non-segmented, positive-sense RNA genome approximately 15 kb in length. The PRRSV genome encodes at least ten open reading frames (ORFs) designated as ORF1a, ORF1b, ORF2a, ORF2b, ORF3, ORF4, ORF5a, ORF5, ORF6, and ORF7 [4-8]. Because PRRSV evolves very quickly [9,10], there is a great deal of genetic variability among PRRSV strains. In general, PRRSV strains are grouped into European (type 1) and North American (type 2) genotypes [11-13], but high levels of genetic variability still exist among viruses, even within the same genotype [10,14,15], which results in suboptimal cross-protection between different PRRSV strains and becomes a significant impediment to the development of effective vaccines. Modified live virus (MLV) vaccines have been most commonly used to control PRRSV because they confer better protection against homologous virus strains compared to inactivated or recombinant vaccines [16]. However, there have been serious safety concerns about using these MLV vaccines because they quickly revert to virulence during serial passages in pigs [17-21]. Therefore, it is important to develop a new strategy to stabilize the PRRSV genome during virus replication for the purpose of vaccine safety.

Previously, a number of nucleoside analogs, including ribavirin (guanosine analog) [22,23], 5-fluorouracil (pyrimidine analog) [24], and 5-azacytidine (cytidine analog) [25], have been shown to be mutagenic, antiviral compounds that are effective against various RNA viruses, such as foot-and-mouth disease virus (FMDV), poliovirus, and hepatitis C virus (HCV). These mutagens increase the mutation frequency of RNA viruses above a tolerable error threshold during replication, ultimately driving viral infection into extinction [26-35]. Amiloride hydrochloride hydrate (hereafter “amiloride”) is another antiviral drug with efficacy against many RNA viruses, including rhinovirus, coxsackievirus B3 (CVB3), coronaviruses, flaviviruses, and retroviruses [36-40]. Moreover, it has been demonstrated that amiloride increases the mutation frequency of RNA viruses, in addition to its other antiviral activities [41]. It has also been demonstrated that mutant viruses that emerged following sequential passages of HCV, CVB3, poliovirus, and FMDV [41-44] in the presence of ribavirin had higher genetic stability than wild-type viruses. Therefore, this strategy was employed to select a mutagen-resistant strain of PRRSV that would have higher genetic stability. In the current study, the effects of four mutagens (ribavirin, 5-fluorouracil, 5-azacytidine, and amiloride) on PRRSV replication in MARC-145 cells were evaluated to select the mutagen that is most effective against PRRSV. Then, mutagen-resistant viruses were rescued after sequential passages in the presence of the mutagen and were evaluated for their genetic stability during additional sequential passages in cell culture systems.

## Methods

### Antiviral mutagens

Ribavirin (Sigma-Aldrich, St. Louis, MO, USA), 5-fluorouracil (Sigma-Aldrich), 5-azacytidine (Sigma-Aldrich), and amiloride (Sigma-Aldrich) were used in this study. All of these mutagens were dissolved in RPMI-1640 medium (Sigma-Aldrich) at stock concentrations of 15 mM (ribavirin) and 20 mM (5-fluorouracil, 5-azacytidine, and amiloride), sterile-filtered using a 0.22-μm syringe filter, aliquoted, and stored at −20°C until use.

### Virus and cell culture

VR2332, a prototype strain of PRRSV type 2, was used in the study. MARC-145, an African Green Monkey kidney cell line highly permissive to PRRSV infection, was used for PRRSV propagation and antiviral assays. MARC-145 cells were maintained in RPMI-1640 medium supplemented with heat-inactivated 10% fetal bovine serum (FBS, Invitrogen, Carlsbad, CA, USA), 2 mM L-glutamine, and 100× antibiotic-antimycotic solution [Anti-anti, Invitrogen; 1× solution contains 100 IU/ml penicillin, 100 μg/ml streptomycin, and 0.25 μg/ml Fungizone® (amphotericin B)] (hereafter referred to as “RPMI growth medium”) at 37°C in a humidified 5% CO_2_ atmosphere.

### Evaluation of effects of mutagens on PRRSV replication

Confluent monolayers of MARC-145 cells were prepared in 25-cm^2^ flasks and were inoculated with VR2332 at a multiplicity of infection (MOI) of 0.01. After incubation for 1 hour in a humidified 5% CO_2_ incubator at 37°C, the virus inoculum was removed, and the cell monolayer was replenished with RPMI growth medium containing one of the antiviral mutagens. Ribavirin, 5-fluorouracil, and amiloride were evaluated at six different concentrations (0, 0.2, 0.4, 0.6, 0.8, and 1 mM), while 5-azactytidine was evaluated at ten different concentrations (0, 0.0001, 0.001, 0.01, 0.1, 0.2, 0.4, 0.6, 0.8, and 1 mM), based on previous reports [45,46]. The treated flasks were then incubated for four more days under the same culture conditions described above, during which time cell culture medium was collected from each flask every 24 hours, centrifuged, and stored at −80°C until analysis.

### Virus titration assay

Progeny virus titers were determined using a microtitration infectivity assay [47]. In brief, up to 8, 10-fold serial dilutions of samples were prepared. Confluent monolayers of MARC-145 cells prepared in 96-well plates were inoculated in quadruplicate with 100 μl of each sample and were incubated for 1 hour under the same culture conditions described above. After incubation, the inoculum was discarded, and the cell monolayer was replenished with RPMI growth medium. The plates were then incubated for an additional six days and monitored for cytopathic effects (CPE) daily. The titer of each virus sample was calculated based on CPE and was expressed as a 50% tissue culture infective dose (TCID_50_)/ml [48].

### Cytotoxicity assay

A commercially available cytotoxicity assay kit (CytoTox-Glo™, Promega, Fitchburg, Wisconsin, USA) was used to assess the cytotoxic effects of the four antiviral mutagens in MARC-145 cells. In short, the CytoTox-Glo™ assay measures a distinct protease (dead-cell protease) activity associated with cytotoxicity [49]. The assay uses a luminogenic peptide substrate (alanyl-alanylphenylalanyl-aminoluciferin; AAF-Glo™ substrate) to measure the activity of dead-cell protease released from cells that have lost membrane integrity. To determine the cytotoxicities of the mutagens, confluent monolayers of MARC-145 cells were prepared in 25-cm^2^ flasks. After rinsing, the cells were replenished with RPMI growth medium containing one of the mutagens at one of four different concentrations (0, 0.5, 1, and 1.5 mM) and further incubated under the culture conditions described above. Supernatants were collected from each flask every 12 hours for up to 48 hours, and the levels of luminescence (RLU) generated from the cleavage of luminogenic AAF-Glo™ substrate by protease in the collected supernatants were measured to determine the cytotoxicity levels induced by each mutagen according to the manufacturer’s instructions.

### Serial passages of PRRSV in MARC-145 cells in the presence of ribavirin

VR2332 was serially passaged in cell culture in the presence of 0, 0.05, 0.1, 0.2, 0.3, 0.5, or 0.7 mM ribavirin to study the emergence of ribavirin-resistant PRRSV mutants. Confluent monolayers of MARC-145 cells were prepared in 6-well plates and pre-treated with RPMI growth medium containing ribavirin for 7 hours prior to infection at 37°C. After the pre-treatment incubation, cells were inoculated with VR2332 at an MOI of 0.01. After incubation for 1 hour, virus inoculum was removed, and cells were replenished with RPMI growth medium containing the same concentrations of ribavirin as described above for post-treatment. The infection was then allowed to proceed for 24 hours, after which the cell culture fluid was collected from each well, centrifuged, and stored at −80°C until use. The supernatant from each passage became the inoculum for the next passage. This procedure was repeated a total of 22 times.

### Assessment of growth kinetics for ribavirin-resistant mutants in the presence of ribavirin at several concentrations using multi-step growth curve analysis

The growth competencies of two ribavirin-resistant candidate mutants (RVRp13 and RVRp22) were assessed in MARC-145 cells in the presence of ribavirin, compared to VR2332 in the presence of ribavirin. Confluent monolayers of MARC-145 cells were prepared in 25-cm^2^ flasks, inoculated with each virus at an MOI of 0.01, and incubated for 1 hour under the same conditions described above. After incubation, the inoculum was discarded, and cells were replenished with RPMI-1640 growth medium containing several concentrations (0, 0.1, 0.2, 0.3, 0.4, and 0.5 mM) of ribavirin. The treated flasks were then incubated for 4 more days. Supernatants were collected from each flask every 24 hours, and the virus in these supernatants was titered.

### Assessment of genetic stability of ribavirin-resistant mutants during passages in MARC-145 cells

To assess the genetic stability of the ribavirin-resistant mutants (RVRp13 and RVRp22) that arose from sequential passages of VR2332 in the presence of ribavirin as described above, the mutant viruses were passaged 10 more times, along with VR2332. Plaque purification was performed using each strain to achieve a highly homogenous virus clone, as described previously [17]. For each passage, confluent MARC-145 cell monolayer prepared in 6-well plates were inoculated with each virus strain and incubated for 1 hour. After incubation, the cells were replenished with RPMI growth medium and incubated for 24 hours. Then, supernatants were collected and used for the next passage of cells. This procedure was repeated 10 times.

Plaque purification was conducted on the supernatants collected after 10 passages to isolate 15 plaque clones per viral strain. Viral RNA was extracted from each virus clone using a commercial kit (Ribo_spin vRD™, GeneAll, Seoul, South Korea) according to the manufacturer’s instructions. nsp2 and ORF5, which are known to be the most variable regions in the PRRSV genome [12,14,50-54], were amplified with a one-step RT-PCR kit (Takara Bio Inc., Otsu, Shiga, Japan) and were submitted for sequencing (Macrogen Inc., South Korea). PCR amplification and sequencing primers are shown in Table 1.

**Table 1:** **Sequences of primers used for PCR amplification and sequencing of the nsp2 and ORF5 regions in the VR2332 genome**

**Sequenced region**	**Primer name**	**Nucleotide location** ^**a**^	**Sequences (5′-3′)**	**Sequenced length (bp)**
nsp2	_p_nsp2F	1249-1268	CCTCCTCAGAATAAGGGTTG	3588
	_p_nsp2R	5120-5138	TGTCAAGGGCAGGGTAAG	
	1a 1481R	1463-1481	GGGAGTAGTGTTTGAGGTG	
	1a 1366F	1366-1383	CTCTTGTGCGACTGCTAC	
	1a 2115R	2097-2115	TACAGGTCAATCTTTGCTG	
	1a 2058F	2058-2075	CCCAGAACAAAACCAACC	
	1a 2867R	2850-2867	ATTGCGGTGAGGACACAA	
	1a 2771F	2771-2788	TGGGAAGATTTGGCTGTT	
	1a 3581R	3563-3581	CAATGGTAAGGTCGCTCTC	
	1a 3511F	3511-3529	TCCGTGTGAGTTTGTGATG	
	1a 4276R	4258-4276	CAGTAACCTGCCAAGAATG	
	1a 4141F	4141-4158	CGCTGCTTGTGAGTTTGA	
ORF5	_p_P5F^r^	13716-13734	CCTGAGACCATGAGGTGGG	603
	_p_P5R^r^	14457-14479	TTTAGGGCATATATCATCACTGG	

a: Location of primers in the full-length VR2332 genome (GenBank accession [AY150564]). p: primers used for PCR amplification and sequencing. The remaining primers were used only for sequencing. r: reference primers used in a previous study [17].

### Data analysis

The effects of the four mutagens on PRRSV replication were analyzed by repeated measures analysis of variance (ANOVA). The Wilcoxon signed-rank test was used to compare the mutation rates of the ribavirin-resistant mutants with that of their parental virus strain. Nucleotide sequences were aligned and analyzed using Lasergene® MagAlign software (DNASTAR Inc., Madison, WI, USA).

## Results

### Effects of antiviral mutagens on in vitro PRRSV replication

**Figure 1:**
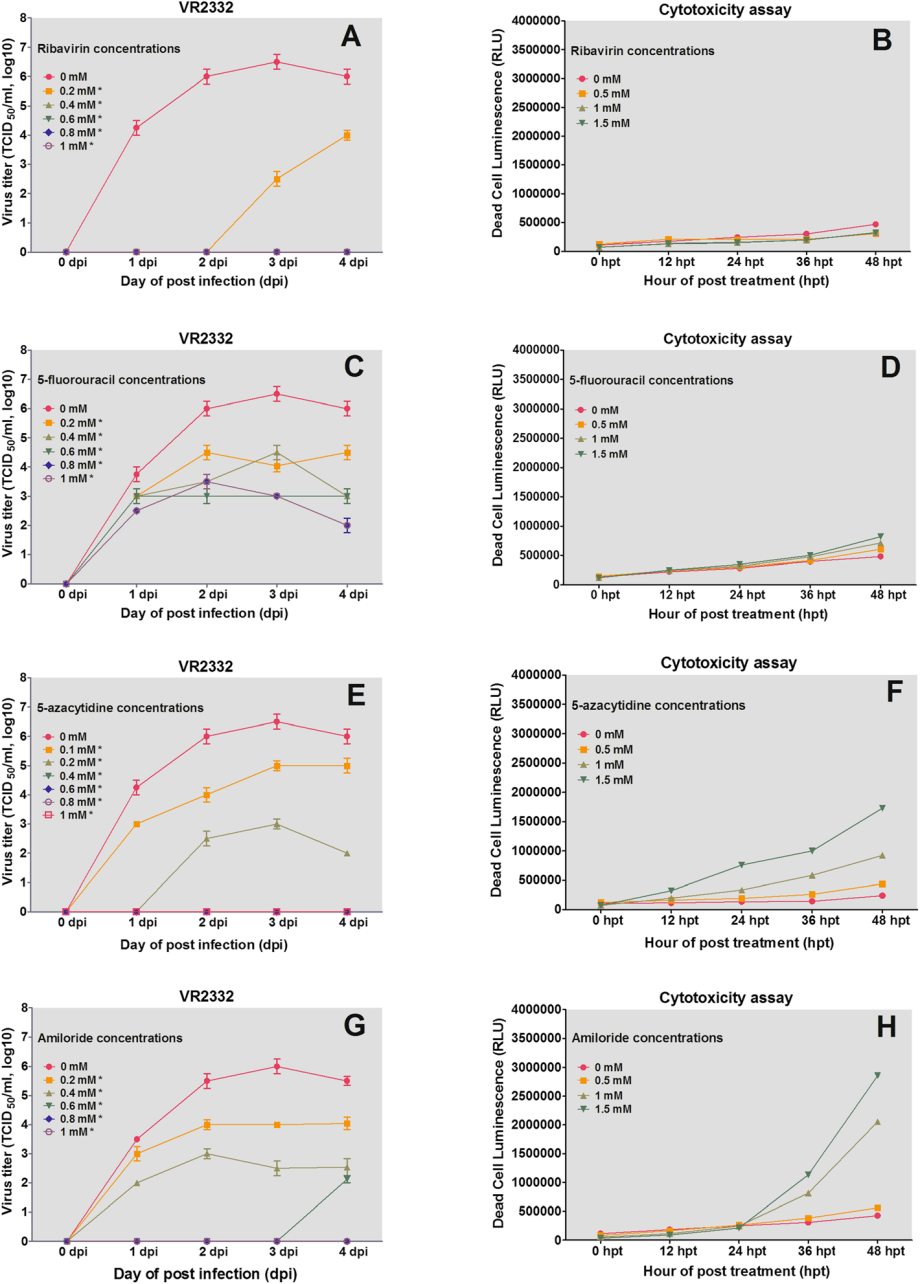
**Evaluation of the effects of four antiviral mutagens on PRRSV replication.** The effects of different concentrations of ribavirin **(A)**, 5-fluorouracil **(C)**, 5-azacytidine **(E)**, and amiloride **(G)** in RPMI-1640 medium on the replication of the PRRSV isolate VR2332 in MARC-145 cells were evaluated, as determined by the production of progeny viruses (TCID_50_/ml) over time. Cytotoxicity assays were performed using cell culture fluids collected from MARC-145 cells every 12 hours after being incubated with the following mutagens: ribavirin **(B)**, 5-fluorouracil **(D)**, 5-azacytidine **(F)**, and amiloride **(H)**, as indicated in the panel. The results are expressed as the luminescence (RLU) from dead-cell protease activity. Asterisks represent a significant difference (*p* < 0.05) in virus replication after mutagen treatment compared to that after vehicle treatment.

The effects of the four antiviral mutagens studied on PRRSV replication are summarized in Figure 1. The rate of VR2332 replication in MARC-145 cells decreased more than 100-fold in the presence of 0.2 mM ribavirin and was completely suppressed at concentrations of ribavirin higher than 0.2 mM. Despite the efficient antiviral effect of ribavirin at low concentrations, no significant cytotoxicity was observed, even at the highest concentration (1.5 mM) of ribavirin studied, up to 48 hours post-treatment {Figure 1 (A and B)}. Similarly, 5-fluorouracil suppressed VR2332 replication in a dose-dependent manner: the rate of VR2332 replication decreased approximately 100-fold or more than 1000-fold in the presence of 0.2 or 1 mM 5-fluorouracil, respectively. No significant cytotoxicity was observed with up to 1.5 mM 5-fluorouracil {Figure 1 (C and D)}. However, significant levels of cytotoxicity were observed with 1 and 1.5 mM azacytidine at 48 hours post-treatment, although VR2332 replication was significantly suppressed when at least 0.1 mM 5-azacytidine was included in the culture medium {Figure 1 (E and F)}. No significant antiviral activity against VR2332 was measured at low concentrations (<0.1 mM) of 5-azacytidine (data not shown). Similarly, high concentrations (1 and 1.5 mM) of amiloride also caused significant cytotoxicity in MARC-145 cells after 36 hours post-treatment {Figure 1 (H)}; however, VR2332 replication was suppressed by amiloride in a dose-dependent manner and was completely suppressed by more than 0.6 mM amiloride {Figure 1 (G)}. Based on these results, ribavirin was selected for further experiments because it was most effective at suppressing PRRSV replication without causing significant cytotoxicity in MARC-145 cells.

### Emergence of ribavirin-resistant mutants after serial passage of PRRSV in MARC-145 cells in the presence of ribavirin

**Figure 2:**
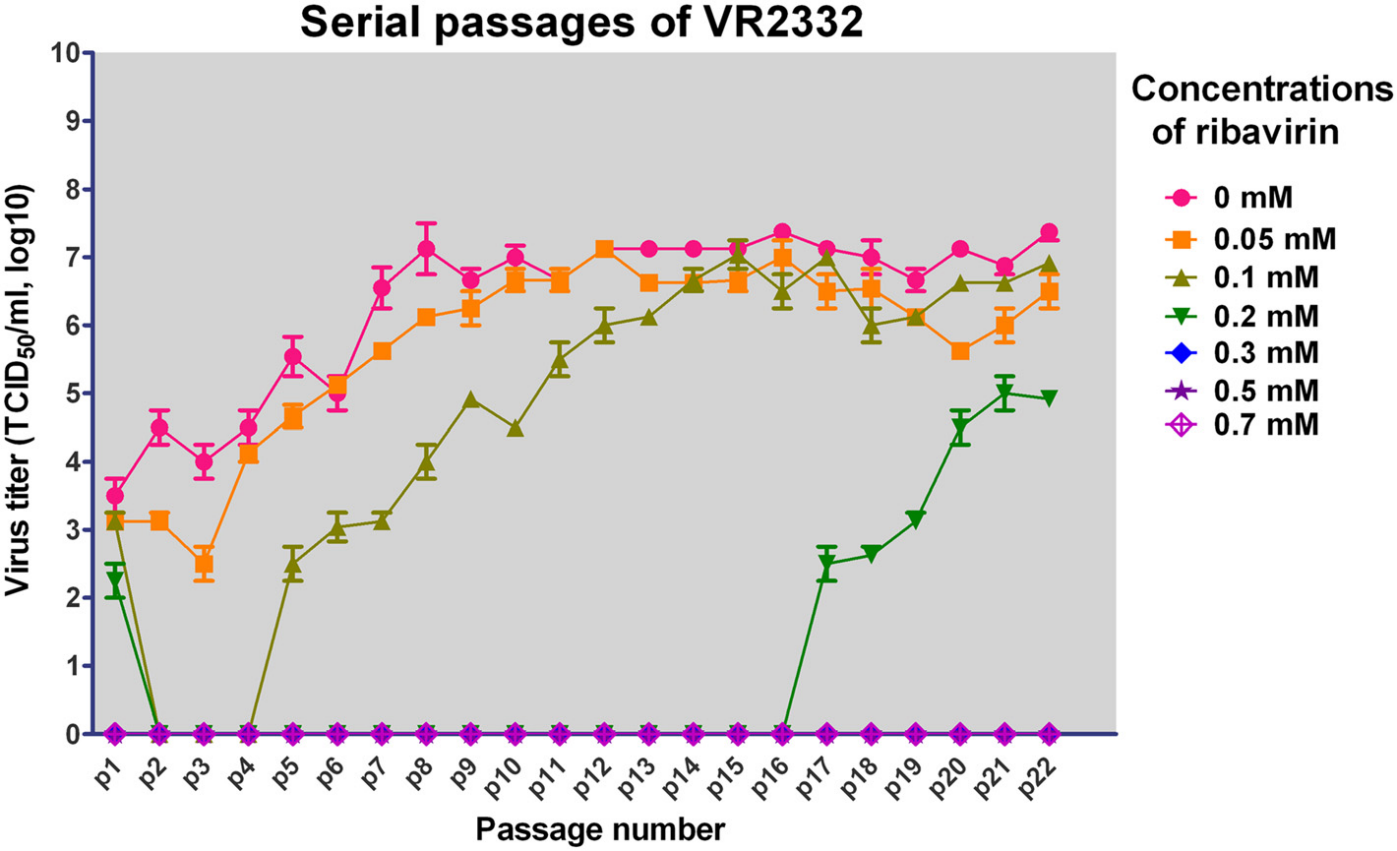
**Emergence of ribavirin-resistant PRRSV mutants during sequential passages with ribavirin in MARC-145 cells.** Ribavirin-resistant mutants emerged during serial passages of the PRRSV isolate VR2332 in MARC-145 cells in the presence of different concentrations of ribavirin, based on the presence or absence of detectable infectious progeny virus at the end of a 24-hour incubation.

VR2332 was serially passaged in MARC-145 cells in the presence of ribavirin at concentrations of 0, 0.05, 0.1, 0.2, 0.3, 0.5, and 0.7 mM. Although 0.05 mM ribavirin failed to mediate significant suppression of VR2332 replication, concentrations of 0.1 and 0.2 mM ribavirin were able to suppress virus replication to undetectable levels based on a virus titration assay at passage 2. However, virus replication started to resume at passages 5 and 17 in the presence of 0.1 and 0.2 mM ribavirin, respectively. The reemerging viruses maintained increasing progeny virus production in successive passages, and two virus strains, RVRp13 and RVRp22, were recovered at passages 13 and 22 in the presence of 0.1 and 0.2 mM ribavirin, respectively. Ribavirin doses greater than or equal to 0.3 mM completely suppressed the replication of VR2332 below the detection limit of the virus titration assay for all 22 passages (Figure 2).

### Growth kinetics of ribavirin-resistant mutants in the presence of ribavirin at several concentrations

**Figure 3:**
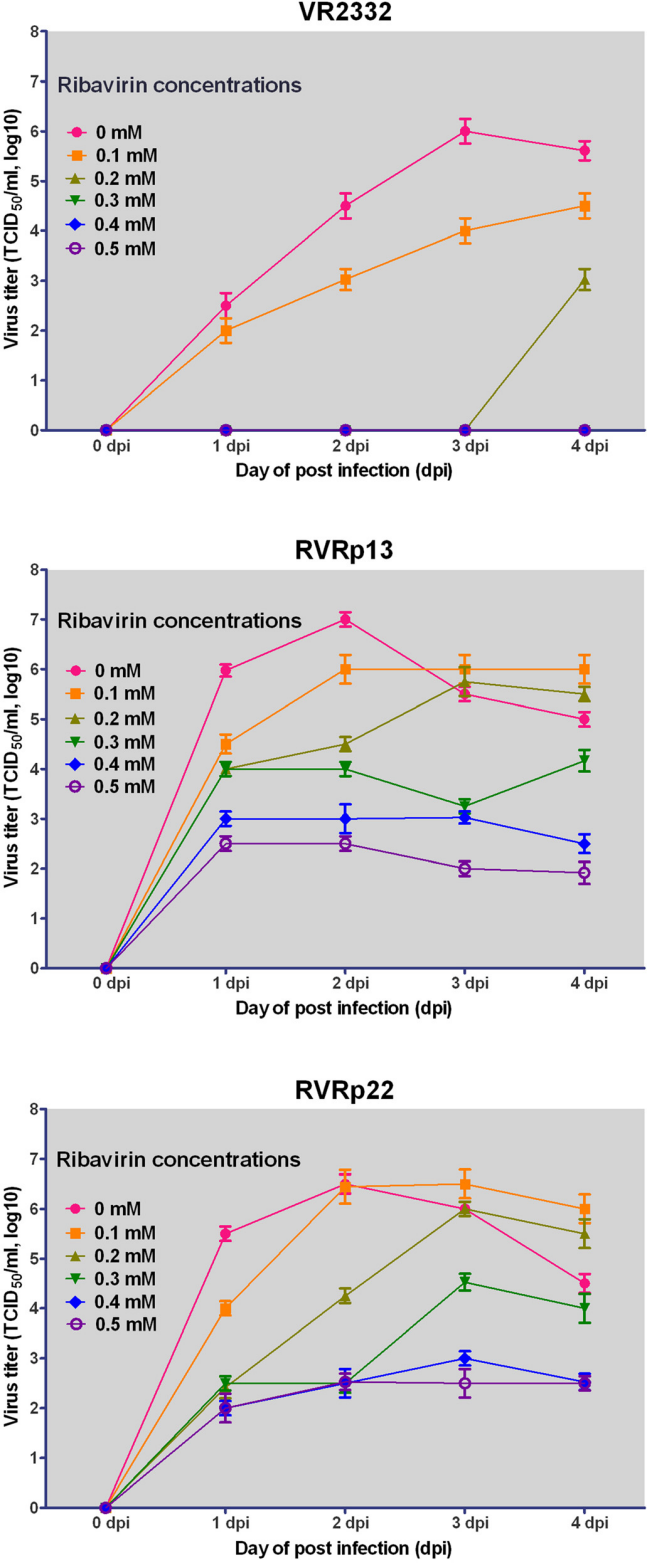
**Assessment of the growth kinetics of ribavirin-resistant mutants in the presence of ribavirin.** The growth kinetics of two ribavirin-resistant PRRSV mutants (RVRp13 and RVRp22) were evaluated in MARC-145 cells in the presence of several concentrations of ribavirin compared to their parental strain VR2332.

RVRp13 and RVRp22 were evaluated for their resistance to ribavirin by assessing their growth competence in MARC-145 cells in the presence of several concentrations (0, 0.1, 0.2, 0.3, 0.4, and 0.5 mM) of ribavirin, compared to the growth competence of the parental virus, VR2332. Both mutant virus strains exhibited higher replication efficiency than did VR2332 in the presence of ribavirin (Figure 3); RVRp13 and RVRp22 both replicated over 10- or 100-times more efficiently than VR2332 in the presence of 0.1 or 0.2 mM ribavirin, respectively. Moreover, VR2332 was unable to replicate in the presence of ribavirin at concentrations of 0.2 mM or higher, whereas RVRp13 and RVRp22 were able to grow to a moderate extent in the presence of ribavirin at concentrations as high as 0.5 mM.

### Higher genetic stability of ribavirin-resistant mutants after serial passages in MARC-145 cells

**Figure 4:**
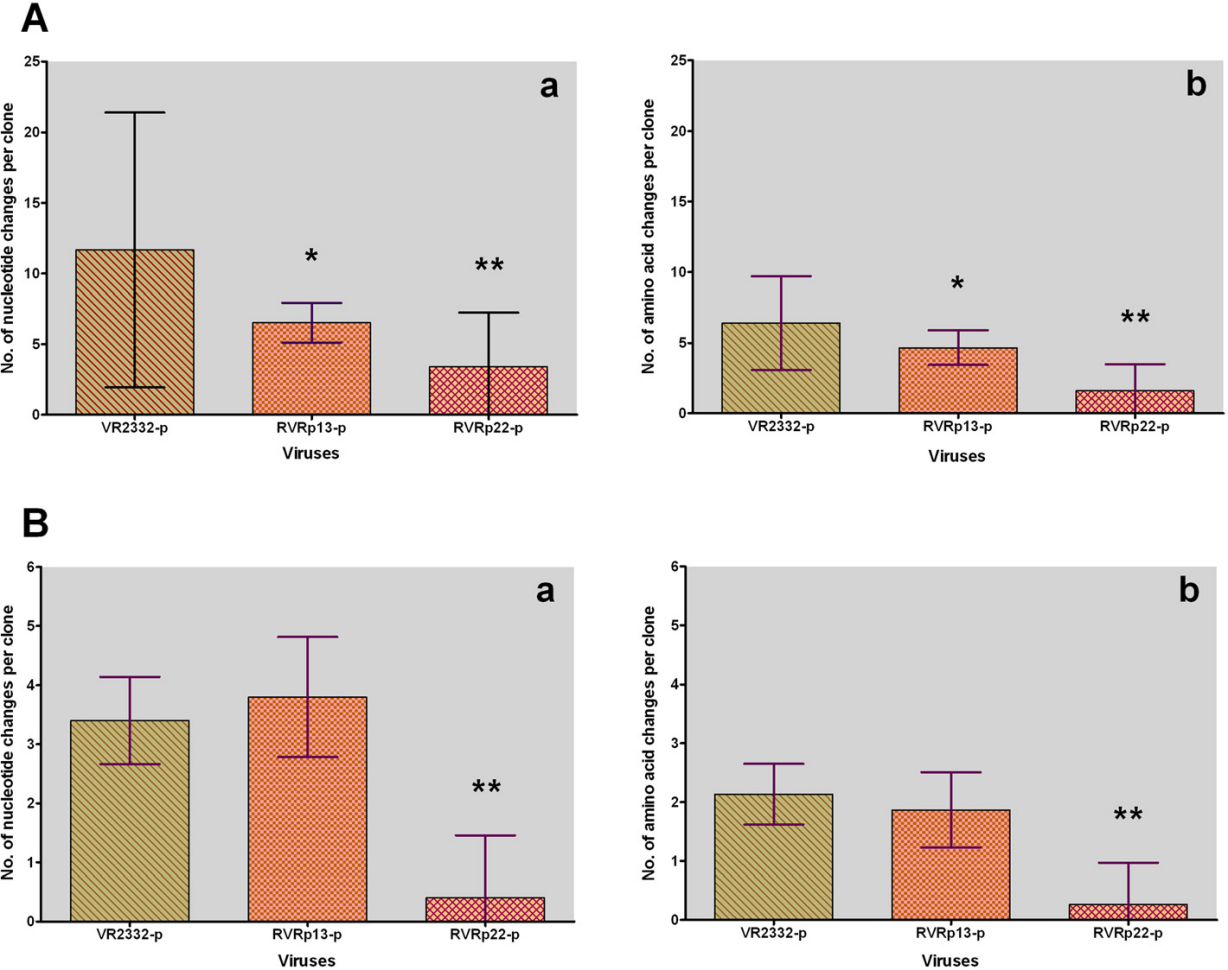
***In vitro*** **assessment of the genetic stability of ribavirin-resistant PRRSV mutants during serial passages in cells.** The genetic stability of the plaque-purified, ribavirin-resistant mutants (RVRp13-P and RVRp22-P) was compared with that of plaque-purified VR2332 (VR2332-p) over 10 passages in MARC-145 cells. Fifteen viral clones obtained from cell culture fluids collected at the completion of 10 passages of each virus by plaque purification and nsp2 **(A)** and ORF5 **(B)** regions of each virus clone were sequenced and compared with their parental viruses (RVRp13-P, RVRp22-P, and VR2332-p) to determine nucleotide mutations **(a)** and amino acid **(b)** substitutions during the sequential passages. Asterisks represent significant differences in the mutation rate compared with VR2332 (* *p* < 0.05, ** *p* < 0.001).

The ribavirin-resistant mutants, RVRp13 and RVRp22, and VR2332 were plaque-purified and designated as RVRp13-p, RVRp22-p, and VR2332-p, respectively. The plaque-purified viruses were then serially passaged 10 times in MARC-145 cells without ribavirin. After 10 passages, 15 virus clones were rescued from each supernatant by plaque purification for each virus strain, and the hypervariable regions (nsp2 and ORF5) of the 15 virus clones were amplified for sequencing. As summarized in Table 2 and Figure 4, RVRp13-p and RVRp22-p exhibited lower mutation frequencies than VR2332-p after 10 sequential passages in MARC-145 cells: 175 nucleotide and 96 amino acid substitutions were identified in the nsp2 region of VR2332-p, whereas RVRp13-p had 98 nucleotide (*p* < 0.05) and 70 amino acid substitutions (*p* < 0.05) and RVRp22-p had 51 nucleotide (*p* < 0.001) and 24 amino acid substitutions (*p* < 0.001) in the same region {Figure 4 (A)}. In ORF5, both RVRp13-p and VR23323-p had similar mutation frequencies, with 57 nucleotide and 29 amino acid substitutions for RVRp13-p and 51 nucleotide and 32 amino acid substitutions for VR23323-p. In contrast, RVRp22-p had a much lower (*p* < 0.001) mutation frequency, accumulating only 6 nucleotide and 4 amino acid substitutions {Figure 4 (B)}.

**Table 2:** **Mutation frequencies of plaque-cloned, ribavirin-resistant mutants (RVRp13-P and RVRp22-P) and VR2332-p after 10 passages in MARC-145 cells**

**Sequenced region**		**VR2332-p**	**RVRp13-p**	**RVRp22-p**
nsp2	Total no. of clones sequenced^a^	15	15	15
	Total no. of nucleotides sequenced (3588 nt per clone)	53820	53820	53820
	Total no. of mutations	175	98	51
	Total no. of nucleotide deletions in sequenced length	63	0	6
	Mutation rate/10^3^ nt	3.25	1.82^*^	0.94^**^
	Total no. of amino acids sequenced (1196 aa per clone)	17940	17940	17940
	Total no. of mutations	96	70	24
	Total no. of amino acid deletions in sequenced length	21	0	2
	Mutation rate/10^3^ aa	5.35	3.90^*^	1.33^**^
ORF5	Total no. of clones sequenced	15	15	15
	Total no. of nucleotides sequenced (603 nt per clone)	9045	9045	9045
	Total no. of mutations	51	57	6
	Mutation rate/10^3^ nt	5.64	6.30	0.66^**^
	Total no. of amino acids sequenced (201 aa per clone)	3015	3015	3015
	Total no. of mutations	32	29	4
	Mutation rate/10^3^ aa	10.61	9.61	1.32^**^

a: The numbers of nucleotide mutations and amino acid substitutions were determined by sequencing 15 plaque-purified virus clones from cell culture fluids collected at the completion of 10 passages of each virus and comparing those samples to the original viruses not submitted to sequential cell passages.

nt: nucleotide, aa: amino acid, significance levels when comparing a drug-resistant strain with VR2332 are indicated by asterisks: *p < 0.05, **p < 0.001*.*

## Discussion

In the current study, the possibility of rescuing a genetically stable PRRSV mutant during sequential passages in MARC-145 cells in the presence of mutagens was explored on the basis of previous reports that showed that mutant viruses that emerged from lethal mutagenesis driven by antiviral mutagens exhibited higher genetic stability than wild-type viruses [35,41,43]. To choose the most appropriate mutagen for the study, four different antiviral mutagens (ribavirin, 5-fluorouracil, 5-azacytidine, and amiloride) were evaluated for their antiviral effects against PRRSV and for their cytotoxicity in MARC-145 cells. In the presence of ribavirin at concentrations higher than 0.2 mM, the replication of VR2332 was completely suppressed, whereas even at the highest concentration, 1.5 mM, ribavirin did not cause significant cytotoxicity in MARC-145 cells {Figure 1 (A and B)}. This result is in good agreement with previous studies conducted to evaluate the antiviral effects of ribavirin on several RNA viruses, including HCV, respiratory syncytial virus (RSV), poliovirus, FMDV, and Influenza A and B viruses [28,30,32,33,42,55-57]. Recently, ribavirin was reported as a potential antiviral drug against PRRSV because it reduced virus replication approximately 100 times in PAM-pCD163 cells when added at a concentration of 0.05 mM, which was the highest concentration applied in the study [58]. However, in the current study, 0.05 mM ribavirin failed to suppress PRRSV replication in MARC-145 cells, and concentrations higher than 0.2 mM ribavirin were required for complete suppression of PRRSV replication (Figure 2). Unlike the results observed in PAM-pCD163 cells, no significant cytotoxicity was observed in MARC-145 cells, even when treated with 1.5 mM ribavirin. Thus, ribavirin-resistant mutant viruses were rescued in the presence of ribavirin at concentrations as high as 0.1 and 0.2 mM. Similarly, 5-fluorouracil effectively suppressed VR2332 replication in MARC-145 cells at concentrations ranging from 0.2 to 1 mM without causing significant cytotoxicity {Figure 1 (C and D)}. The effective antiviral activity of 5-fluorouracil has also been reported for rift valley fever virus, vesicular stomatitis virus, poliovirus, and FMDV [26,59-63]. In contrast, 5-azacytidine or amiloride at concentrations between 1 and 1.5 mM induced significant cytotoxicity in MARC-145 cells, although concentrations of 5-azacytidine or amiloride lower than 1 mM showed substantial antiviral efficacy against PRRSV {Figure 1 (E and F) and Figure 1 (G and H)}*.* A previous study reported that 1 mM 5-azacytidine caused significant cytotoxicity in 293 T and U373-MAGI_CXCR4_ cells 24 hours after treatment [46] although a significant level of antiviral activity against HIV-1 was reported with 5-azacytidine at concentrations between 1 μM and 100 μM [45,46]. However, low concentrations (<0.1 mM) of 5-azacytidine did not result in significant antiviral activity against PRRSV in the current study (data not shown). A previous study reported that 1 mM amiloride was non-cytotoxic in HeLa T cells for 48 hours post-treatment [38], which conflicts with the results observed in this study showing that substantial cytotoxicity was observed with 1–1.5 mM amiloride in MARC-145 cells at 36 hours post-treatment {Figure 1 (H)}. It was speculated that this dissimilarity might be due to the different origins of the cell lines used in the studies.

Based on the initial assessment of antiviral mutagens, ribavirin was selected for further study to evaluate the possible emergence of ribavirin-resistant mutants because it showed the greatest effect on PRRSV replication without causing significant cytotoxicity even at the highest concentration (1.5 mM). Two ribavirin-resistant mutants (RVRp13 and RVRp22) were isolated at passages 13 and 22 during serial passages of VR2332 in MARC-145 cells in the presence of 0.1 and 0.2 mM ribavirin, respectively (Figure 2). Previous studies also reported the emergence of ribavirin-resistant mutants: ribavirin-resistant HCV emerged after 7 passages in Huh7D cells cultured with 0.25 mM ribavirin [42]. Similarly, ribavirin-resistant CVB3, poliovirus, and FMDV emerged after sequential passages in HeLa or BHK-21 cells cultured with 0.05-0.8 mM ribavirin [41,43,44].

The growth competence of the ribavirin-resistant mutants RVRp13 and RVRp22 in MARC-145 cells was assessed in the presence or absence of ribavirin. Both of the ribavirin-resistant mutants showed approximately 10-100-times higher replication efficiency than VR2332 in the presence of 0.1 or 0.2 mM ribavirin. Moreover, the replication of VR2332 was completely suppressed at concentrations higher than 0.2 mM ribavirin, while both mutants were able to generate infectious progeny viruses, reaching titers close to 10^3^ TCID_50_/ml in the presence of 0.5 mM ribavirin (Figure 3). As reported in many previous studies conducted with poliovirus [43], human enterovirus 71 (HEV71) [64], FMDV [65,66], coxsackie virus B3 [41] and HCV [42], enhanced resistance of mutant viruses to ribavirin might be associated with the increased genetic fidelity that is acquired during viral passages in the presence of ribavirin. However, the higher replication efficiency might also result from virus adaptation to MARC-145 cells because the mutant viruses were rescued after 13 or 22 sequential passages. In fact, the mutant viruses replicated more efficiently in the absence of ribavirin compared to VR2332. Therefore, the genetic stability of the ribavirin-resistant mutants was assessed by passaging the mutant viruses 10 times in MARC-145 cells without ribavirin in parallel with VR2332 to demonstrate that ribavirin-resistant mutants have increased genetic stability. The most variable genes (nsp2 and ORF5) in the PRRSV genome were sequenced to determine the mutation frequency of the viruses during sequential passages. RVRp13 and RVRp22 virus clones exhibited 1.8- and 3.4-fold lower numbers of nucleotide substitutions and 1.4- and 4-fold lower numbers of amino acid substitutions, respectively, in the nsp2 region compared with VR2332 virus clones {Table 2 and Figure 4 (A)}. In the ORF5 region, the RVRp13 virus clones exhibited mutation rates similar to those observed in VR2332 virus clones, while RVRp22 virus clones had approximately 8.5-fold lower numbers of nucleotide substitutions and 8-fold lower numbers of amino acid substitutions compared with VR2332 virus clones {Table 2 and Figure 4 (B)}. Based on these results, it was concluded that the ribavirin-resistant mutants, especially RVRp22, have significantly higher genetic stability compared with their parental virus, VR2332.

## Conclusions

In conclusion, ribavirin was very effective in suppressing PRRSV replication in MARC-145 cells at concentrations higher than 0.2 mM, suggesting that ribavirin could be used as a therapeutic drug against PRRSV; however, its potential usefulness against PRRSV infection remains to be confirmed in animal studies. As described in the current study, the resistant viruses emerged in the presence of low concentrations (<0.2 mM) of ribavirin, and those resistant viruses had significantly higher genetic stability compared with VR2332. Because rapid reversion of attenuated PRRS vaccines to virulence is of great concern, RVRp22, which has a higher level of genetic stability, could be a good candidate for the development of a safer vaccine. Nonetheless, the mechanisms and genetic determinants responsible for the high genetic stability of ribavirin-resistant PRRSV should be elucidated in detail in the near future.

## Abbreviations

PRRSV: Porcine reproductive and respiratory syndrome virus
MLV: Modified live virus
EAV: Equine arteritis virus
LDV: Lactate dehydrogenase-elevating virus of mice
SHFV: Simian hemorrhagic fever virus
ORF: Open reading frame
FMDV: Foot-and-mouth disease virus
HCV: Hepatitis C virus
CVB3: Coxsackievirus B3
FBS: Fetal bovine serum
MOI: Multiplicity of infection
CPE: Cytopathic effect
TCID: Tissue culture infective dose
AAF: Alanyl-alanylphenylalanyl-aminoluciferin
RLU: Relative luminescence units
nsp2: Nonstructural protein 2, RSV, Respiratory syncytial virus
RVF: Rift valley fever virus
VSV: Vesicular stomatitis virus
HIV-1: Human immunodeficiency virus 1
